# Low systemic vascular resistance with normal blood pressure: Do we need vasopressors?

**DOI:** 10.1371/journal.pone.0333365

**Published:** 2025-10-03

**Authors:** Min Woo Kang, Shin Young Ahn, Yoonjin Kang

**Affiliations:** 1 Department of Internal Medicine, Korea University Guro Hospital, Seoul, Korea; 2 Department of Thoracic and Cardiovascular Surgery, Seoul National University Hospital, Seoul, Korea; 3 Seoul National University, College of Medicine, Seoul, Korea; Mayo Clinic College of Medicine and Science, UNITED STATES OF AMERICA

## Abstract

**Background:**

Vasoplegia is defined by systemic vascular resistance (SVR) < 800 dynes·s/cm^5^, cardiac index (CI) > 2.2 L/min/m^2^, and hypotension, and is associated with poor outcomes. Maintaining adequate blood pressure with vasopressors is considered essential in these cases. However, some patients exhibit low SVR and high CI while maintaining normal blood pressure, and the benefits of vasopressor use in this population have not been studied.

**Methods:**

This study utilized the Medical Information Mart for Intensive Care-IV (MIMIC-IV) database and included intensive care unit (ICU) patients who met the criteria for vasoplegic status, defined as SVR < 800 dynes·s/cm^5^, CI > 2.2 L/min/m^2^, and mean arterial pressure (MAP) ≥ 60 mmHg. The primary outcome was kidney deterioration, and the secondary outcome was prolonged ICU stay (defined as > 3 days). Firth’s logistic regression was used to analyze associations. Subgroup analyses and interaction term p-values were also assessed to evaluate the modifying effects of relevant variables on the relationship between vasopressor use and the outcomes.

**Results:**

Among 319 patients, 6.0% experienced kidney deterioration, and 24.1% had a prolonged ICU stay. Vasopressor use was not significantly associated with kidney deterioration (odds ratio [OR]: 2.96 [0.33–29.81], p = 0.321); however, it was significantly associated with prolonged ICU stay (OR: 4.71 [2.15–10.77], p < 0.001). Sensitivity analyses confirmed these findings across vasopressor types. Subgroup analysis showed that vasopressor use was associated with a higher risk of prolonged ICU stay in patients without congestive heart failure, without heart surgery, and without chronic kidney disease.

**Conclusions:**

In patients with low SVR and normal blood pressure, vasopressor use was associated with longer ICU stays but not kidney protection.

## Introduction

Vasoplegia is defined as a condition characterized by a systemic vascular resistance (SVR) of less than 800 dynes·s/cm^5^ and a cardiac index (CI) greater than 2.2 L/min/m^2^, accompanied by hypotension, most commonly defined as a mean arterial pressure (MAP) < 60 [1,2]. It reflects a state of refractory vasodilation driven by endothelial dysfunction, dysregulated inflammatory mediator release (e.g., nitric oxide, interleukin-6, interleukin-8), and reduced vascular responsiveness to catecholamines [1,2]. This phenomenon is especially prevalent after cardiovascular surgery—particularly cardiopulmonary bypass—where contact activation, ischemia–reperfusion injury, and systemic inflammation further impair endothelial tone and amplify vasodilatory mediator release; it is also common in severe infections such as septic shock [1,3,4]. Vasoplegia is believed to be triggered by an inflammatory response, with key mechanisms involving the release of cytokines such as interleukin-6, interleukin-8, and bradykinin, as well as endothelial damage [5–7]. The incidence of vasoplegia in patients undergoing cardiac surgery is reported to be between 10% and 30% [4,8], and it is associated with severe complications, including prolonged ICU stay, increased transfusion requirements, kidney injury, and increased mortality [4,9–11].

Vasoplegia is characterized by persistent, uncontrolled dilation of the vasculature, leading to reduced organ perfusion and consequent kidney injury and other end-organ damage. To prevent this, maintaining adequate blood pressure is crucial, and vasopressors, particularly norepinephrine and vasopressin, have been commonly used for this purpose [12–14]. While these treatments help stabilize blood pressure and protect organ function, excessive use of vasopressors can lead to adverse effects, necessitating careful management [15,16].

Blood pressure targets for vasoplegia typically aim for a mean arterial pressure (MAP) of 60–70 mmHg [17,18]. However, a subgroup of critically ill patients exhibits hallmark features of vasoplegia—SVR below normal and CI above expected values—while maintaining MAP ≥ 60 mmHg. Although not encompassed by traditional shock definitions, these normotensive vasodilated patients suffer from pathological vasodilation. This may reflect compensatory rises in cardiac output—via reflex tachycardia, increased contractility, and augmented venous return—that offset reduced vascular resistance and preserve arterial pressure. Evidence regarding the efficacy and safety of vasopressor therapy in this setting is scarce, with limited data on its effects on organ perfusion, renal outcomes, and ICU length of stay, because clinical studies have traditionally focused on hypotensive shock and have overlooked normotensive vasodilated phenotypes. By increasing systemic vascular resistance and mean arterial pressure, vasopressors may theoretically improve renal blood flow yet also carry risks—such as excessive vasoconstriction and drug-related adverse events—that could prolong ICU recovery. In this study, we define this condition as “vasoplegic status” and investigate the association between vasopressor administration, kidney deterioration, and prolonged ICU stay in critically ill patients. We focus on a normotensive subset—patients exhibiting SVR < 800 dyn·s·cm⁻⁵ and CI > 2.2 L/min/m^2^ while maintaining MAP ≥ 60 mmHg—whom we designate as having “vasoplegic status.” Although this group falls outside the conventional shock definition, they experience similar pathophysiologic vasodilation, and the risks and benefits of vasopressor therapy in this setting remain poorly characterized.

## Methods

### Ethical approval

As this study was an analysis of a public database, this study was exempt from approval by the institutional review board of Seoul National University Hospital (no. 2405-061-1535).

### Study population

This study is a retrospective cohort study utilizing the Medical Information Mart for Intensive Care (MIMIC)‑IV database [19]. MIMIC-IV is a large, publicly available database containing de-identified patient data collected from the ICUs at Beth Israel Deaconess Medical Center. We used version 3.0 of the MIMIC-IV database, which includes data from 2008 to 2022 and contains over 94,000 ICU patient records.

From this dataset, we excluded patients with underlying end-stage kidney disease or those receiving renal replacement therapy at the time of ICU admission. Additionally, only patients who met the criteria for vasoplegic status, defined as SVR < 800 dynes·s/cm^5^ and CI > 2.2 L/min/m^2^, but who maintained a MAP ≥ 60 mmHg (i.e., those without hypotension) were included in the analysis. Therefore, only patients whose initial SVR and CI at the time of ICU admission met these criteria, and whose lowest MAP value within the first 24 hours of ICU admission was ≥ 60 mmHg, with corresponding SVR and CI values also meeting the aforementioned criteria, were included in the analysis.

### Exposures and outcomes

The exposure variable in this study was the administration of vasopressors within the first 24 hours after ICU admission. The types of vasopressors examined included norepinephrine, vasopressin, epinephrine, and dopamine. For the purpose of sensitivity analysis, the use of vasopressors was further categorized. Since norepinephrine and vasopressin are the most commonly used drugs in vasoplegia treatment, we classified the patients into four groups based on the administration of these drugs: 1) administration of norepinephrine or vasopressin without epinephrine, 2) administration of norepinephrine or vasopressin in combination with epinephrine, 3) administration of epinephrine only, and 4) no vasopressor administration. We then analyzed the association of these categories with the outcomes.

The primary outcome was kidney deterioration, defined as a doubling of serum creatinine levels compared to ICU admission levels or the initiation of renal replacement therapy after 24 hours of ICU admission. The secondary outcome was prolonged ICU stay, defined as an ICU stay exceeding 3 days, which has been recognized as a predictor of adverse outcomes following cardiac surgery [20].

### Variables

Initial SVR and CI were defined as the values measured within 24 hours prior to ICU admission and up to 6 hours after admission, using the measurements closest to the time of ICU admission. These measurements were obtained by intermittent bolus thermodilution via Swan–Ganz pulmonary artery catheters and by continuous flow monitoring with Impella devices. SVR and CI at low MAP were defined as the SVR and CI values measured closest to the time when the lowest MAP value was recorded within 24 hours after ICU admission. The variables included in the analysis were vital signs at ICU admission (systolic blood pressure [SBP], diastolic blood pressure [DBP], heart rate, peripheral oxygen saturation [SpO_2_]) and demographic data (age, sex, body surface area [BSA]). Underlying diseases (hypertension, diabetes, chronic kidney disease [CKD], chronic liver disease, congestive heart failure, myocardial infarction, and cerebrovascular disease) were also included, defined by ICD diagnostic codes. Blood test results closest to ICU admission, measured within the period from 24 hours before ICU admission to 6 hours after, were included (creatinine, hemoglobin, lactate, bicarbonate, pH). Baseline creatinine was also included, defined as the lowest creatinine value measured within 3 months prior to ICU admission. Cases in which patients underwent heart or aorta surgery before ICU admission were identified using ICD procedure codes. Additionally, whether mechanical ventilation was administered within 6 hours of ICU admission, and whether atrial fibrillation was present within 24 hours of ICU admission, were also included.

### Statistical analysis

Continuous variables were presented as mean ± standard deviation, while categorical variables were expressed as counts and percentages. Firth’s logistic regression was used to determine the odds ratios (OR) and 95% confidence intervals (CI) for the associations between vasopressor use, kidney deterioration, and prolonged ICU stay duration. Firth’s penalized logistic regression has gained wide acceptance for modeling binary outcomes in small‐sample and rare‐event studies, as its penalized likelihood framework reduces bias and ensures stable estimates in the presence of separation [21]. Both univariable and four multivariable models were utilized. In Model 1, adjustments were made for age, sex, CKD, and creatinine. Model 2 included the variables from Model 1, with additional adjustments for SBP, DBP, heart rate, BSA, heart surgery, aorta surgery, initial SVR, initial CI, SVR at low MAP, and CI at low MAP. Model 3 further adjusted for mechanical ventilation, atrial fibrillation, congestive heart failure, diabetes, and hypertension. Model 4 added further adjustments for pH, bicarbonate, lactate, and hemoglobin to the variables in Model 3. Multicollinearity among predictors was evaluated using variance inflation factors (VIF). Additionally, we assessed model discrimination by calculating the concordance statistic (c-statistic) for each fitted model. Potential effect modifications by advanced age (≥ 65 years), sex, tachycardia (heart rate ≥ 100), congestive heart failure, and CKD were assessed through subgroup analyses and interaction term tests, based on prior research indicating the relevance of these factors to mortality risk [22,23]. Subgroup and interaction results are displayed graphically as forest plots to facilitate comparison across groups

For the sensitivity analysis, the group that received no vasopressors was used as the reference, and the association between vasopressors administration and the outcomes was analyzed using logistic regression, applying the same methodology as described earlier. To further evaluate the stability of the associations between vasopressor administration and clinical outcomes, we replicated the primary logistic regression models, substituting the original mean arterial pressure threshold with an alternative cutoff of 65 mmHg while otherwise preserving all model specifications. All statistical analyses were performed using the latest version of R software (R version 4.1.2). P-values were two-sided, and a significance level of 0.05 was applied.

## Results

### Baseline characteristics

**Fig 1:**
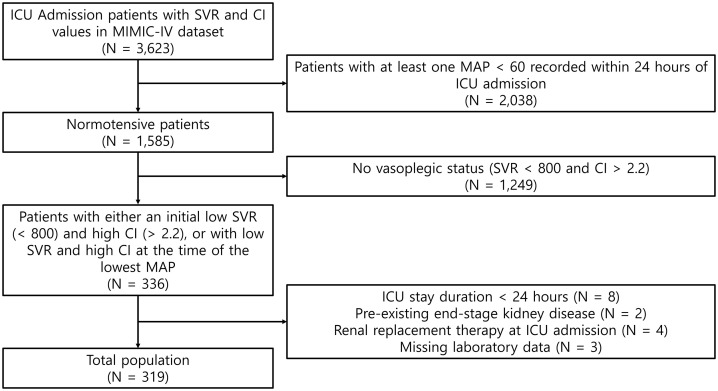
Flow diagram of the study population. ICU, intensive care unit; SVR, systemic vascular resistance; CI, cardiac index; MAP, mean arterial pressure; MIMIC, Medical Information Mart for Intensive Care.

Among the ICU admissions in the MIMIC-IV database, 3,623 patients had recorded measurements of SVR and CI, and 2,038 patients who exhibited a MAP < 60 mmHg at any point after admission were excluded (Fig 1). Additionally, 1,249 patients who did not meet the criteria for vasoplegic status either at the time of ICU admission or at the time of their lowest MAP were excluded. Further exclusions included 8 patients with an ICU stay of less than 24 hours, 2 patients with pre-existing end-stage kidney disease (ESKD), 4 patients receiving renal replacement therapy at ICU admission, and 3 patients with missing laboratory data. This left a total of 319 patients included in the final analysis. When applying a MAP threshold of 65 mmHg for the sensitivity analysis, 136 patients fulfilled the inclusion criteria.

Kidney deterioration occurred in 6.0% of the patients, and 24.1% experienced a prolonged ICU stay. The mean age of the population was 59.8 years, and 91.2% of the patients were male (Table 1). The the mean lowest MAP was 64.6 mmHg. The average ICU stay duration was 70.0 hours. Vasopressors were used in 25.4% of the total population, with 21.1% in the group that developed kidney deterioration and 25.7% in the group that did not, showing no statistically significant difference between the two groups. Among the 319 patients included in the final analysis, none received dopamine within the first 24 hours of ICU admission.

**Table 1:** Baseline characteristics.

Variable	Total (N = 319)	Kidney outcome (N = 19)	No kidney outcome (N = 300)	p-value^*^
Age (years)	59.79 ± 11.29	56.47 ± 12.88	60.00 ± 11.17	0.257
Male	91.2% (291)	73.7% (14)	92.3% (277)	0.018
Heart rate (/min)	83.58 ± 12.05	93.16 ± 13.58	82.97 ± 11.71	0.005
SBP (mmHg)	115.68 ± 16.59	133.21 ± 23.79	114.57 ± 15.43	0.003
DBP (mmHg)	60.18 ± 8.65	66.11 ± 8.00	59.8 ± 8.56	0.003
SpO_2_ (%)	98.88 ± 2.14	99.26 ± 1.24	98.85 ± 2.19	0.200
Body surface area (m^2^)	2.17 ± 0.24	2.14 ± 0.33	2.17 ± 0.23	0.718
Lowest MAP (mmHg)	64.60 ± 4.80	69.95 ± 7.30	64.26 ± 4.40	0.003
Initial SVR (dynes*sec/cm^5^)	861.58 ± 280.89	688.53 ± 200.20	872.54 ± 281.91	0.001
Initial CI (L/min/m^2^)	3.13 ± 1.04	4.47 ± 1.31	3.04 ± 0.97	<0.001
SVR at low MAP (dynes*sec/cm^5^)	718.45 ± 165.24	656.37 ± 148.62	722.38 ± 165.68	0.076
CI at low MAP (L/min/m^2^)	3.36 ± 0.95	4.19 ± 1.06	3.30 ± 0.92	0.002
Hemoglobin (g/dL)	10.65 ± 2.05	9.53 ± 1.45	10.72 ± 2.06	0.003
Lactate (mmol/L)	2.34 ± 1.49	4.94 ± 2.85	2.18 ± 1.18	0.001
Bicarbonate (mmol/L)	23.46 ± 2.71	22.05 ± 3.94	23.55 ± 2.60	0.118
pH	7.37 ± 0.07	7.33 ± 0.09	7.38 ± 0.07	0.036
Creatinine (mg/dL)	0.99 ± 0.40	1.24 ± 1.04	0.97 ± 0.32	0.274
Heart surgery	76.5% (244)	10.5% (2)	80.7% (242)	<0.001
Aorta surgery	4.7% (15)	0% (0)	5% (15)	0.66
Congestive heart failure	32.6% (104)	31.6% (6)	32.7% (98)	>0.999
Hypertension	53.6% (171)	36.8% (7)	54.7% (164)	0.203
Diabetes	29.5% (94)	31.6% (6)	29.3% (88)	>0.999
Chronic kidney disease	11.3% (36)	26.3% (5)	10.3% (31)	0.078
Atrial fibrillation	4.1% (13)	5.3% (1)	4% (12)	>0.999
Mechanical ventilation	89.0% (284)	94.7% (18)	88.7% (266)	0.658
Vasopressors	25.4% (81)	21.1% (4)	25.7% (77)	0.860
Norepinephrine and/or vasopressin	6.9% (22)	10.5% (2)	6.7% (20)	0.859
Epinephrine	13.8% (44)	0% (0)	14.7% (44)	0.146
Norepinephrine and/or vasopressin and epinephrine	4.7% (15)	10.5% (2)	4.3% (13)	0.498
Length of ICU stay (hours)	69.97 ± 123.03	190.9 ± 270.38	62.31 ± 103.49	0.054

Abbreviation: SBP, systolic blood pressure; DBP, diastolic blood pressure; SpO^2^, peripheral oxygen saturation; MAP, mean arterial pressure; SVR, systemic vascular resistance; CI, cardiac index; ICU, intensive care unit.

Note: Kidney outcome is defined as a doubling of serum creatinine compared with ICU admission or initiation of renal replacement therapy after 24 hours.

* Chi-square test for categorical variables and t-test for continuous variables.

### Model C-statistics and collinearity assessment

Across both outcomes, model c-statistics improved steadily from the univariable analysis through to the fully adjusted model. For the kidney outcome, the c‑statistic increased from 0.523 in the univariable model to 0.973 in Model 4, while for prolonged ICU length of stay it rose from 0.572 to 0.821 over the same range (S1 Table). VIF for all predictor variables are shown in S2 Table. VIFs ranged from a minimum of 1.07 (mechanical ventilation) to a maximum of 11.26 (initial cardiac index), with most predictors below 5.

### Association between vasopressors and risk of kidney deterioration

The use of vasopressors was not statistically associated with the occurrence of kidney deterioration in any of the models, from the univariable model through to models 1–4 (Table 2). In the model 1, the odds ratio (OR) was 0.85 (95% CI: 0.24–2.47), with a p-value of 0.777. In model 4, which adjusted for all variables, the OR was 2.96 (95% CI: 0.33–29.81), with a p-value of 0.321. In the sensitivity analysis stratified by vasopressor type, none of the subgroup comparisons reached statistical significance for kidney deterioration (S3 Table).

**Table 2:** Odds ratio for kidney outcome according to using vasopressors.

Group	No. of group	No. of kidney outcome	Univariable		Model 1^a^		Model 2^b^		Model 3^c^		Model 4^d^	
			Odds ratio	p-value	Odds ratio	p-value	Odds ratio	p-value	Odds ratio	p-value	Odds ratio	p-value
No vasopressors	238	15	reference		reference		reference		reference		reference	
Vasopressors	81	4	0.84 (0.25-2.30)	0.744	0.85 (0.24-2.47)	0.777	4.28 (0.79-27.26)	0.092	4.59 (0.57-43.99)	0.158	2.96 (0.33-29.81)	0.321

^a^: Adjusted for age, male, chronic kidney disease, creatinine.

^b^: Adjusted for age, male, systolic blood pressure, diastolic blood pressure, heart rate, body surface area, chronic kidney disease, creatinine, heart surgery, aorta surgery, initial systemic vascular resistance, initial cardiac index, systemic vascular resistance at low mean arterial pressure, cardiac index at low mean arterial pressure.

^c^: Adjusted for age, male, systolic blood pressure, diastolic blood pressure, heart rate, body surface area, chronic kidney disease, creatinine, heart surgery, aorta surgery, initial systemic vascular resistance, initial cardiac index, systemic vascular resistance at low mean arterial pressure, cardiac index at low mean arterial pressure, mechanical ventilation, atrial fibrillation, congestive heart failure, diabetes, hypertension.

^d^: Adjusted for age, male, systolic blood pressure, diastolic blood pressure, heart rate, body surface area, chronic kidney disease, creatinine, heart surgery, aorta surgery, initial systemic vascular resistance, initial cardiac index, systemic vascular resistance at low mean arterial pressure, cardiac index at low mean arterial pressure, mechanical ventilation, atrial fibrillation, congestive heart failure, diabetes, hypertension, ph, bicarbonate, lactate, hemoglobin.

### Association between vasopressors and risk of prolonged ICU stay duration

The use of vasopressors was statistically significantly associated with the occurrence of prolonged ICU stay duration in all models, including the univariable model (Table 3). In the model 1, the OR was 2.02 (95% CI: 1.12–3.59), with a p-value of 0.019. In model 4, the OR increased to 4.71 (95% CI: 2.15–10.77), with a p-value of <0.001, indicating a higher risk of prolonged ICU stay. All vasopressor subgroups remained significantly associated with prolonged ICU stay in the fully adjusted model (S4 Table).

**Table 3:** Odds ratio for prolonged length of intensive care unit stay according to using vasopressors.

Group	No. of group	No. of prolonged LOS	Univariable		Model 1^a^		Model 2^b^		Model 3^c^		Model 4^d^	
			Odds ratio	p-value	Odds ratio	p-value	Odds ratio	p-value	Odds ratio	p-value	Odds ratio	p-value
No vasopressors	238	49	reference		reference		reference		reference		reference	
Vasopressors	81	28	2.04 (1.17-3.53)	0.013	2.02 (1.12-3.59)	0.019	3.30 (1.67-6.68)	0.001	3.54 (1.69-7.62)	0.001	4.71 (2.15-10.77)	<0.001

Abbreviation: LOS, length of stay.

^a^: Adjusted for age, male, chronic kidney disease, creatinine.

^b^: Adjusted for age, male, systolic blood pressure, diastolic blood pressure, heart rate, body surface area, chronic kidney disease, creatinine, heart surgery, aorta surgery, initial systemic vascular resistance, initial cardiac index, systemic vascular resistance at low mean arterial pressure, cardiac index at low mean arterial pressure.

^c^: Adjusted for age, male, systolic blood pressure, diastolic blood pressure, heart rate, body surface area, chronic kidney disease, creatinine, heart surgery, aorta surgery, initial systemic vascular resistance, initial cardiac index, systemic vascular resistance at low mean arterial pressure, cardiac index at low mean arterial pressure, mechanical ventilation, atrial fibrillation, congestive heart failure, diabetes, hypertension.

^d^: Adjusted for age, male, systolic blood pressure, diastolic blood pressure, heart rate, body surface area, chronic kidney disease, creatinine, heart surgery, aorta surgery, initial systemic vascular resistance, initial cardiac index, systemic vascular resistance at low mean arterial pressure, cardiac index at low mean arterial pressure, mechanical ventilation, atrial fibrillation, congestive heart failure, diabetes, hypertension, ph, bicarbonate, lactate, hemoglobin.

### Sensitivity analysis at a MAP threshold of 65 mmHg

Using a MAP cut-off of 65 mmHg, vasopressor use was not significantly associated with kidney deterioration (OR 1.41; 95% CI 0.04–93.22; p = 0.824) (S5 Table). In contrast, vasopressor administration remained strongly associated with prolonged ICU length of stay (OR 4.71; 95% CI 2.15–10.77; p < 0.001) (S6 Table).

### Subgroup analysis and interaction term

**Fig 2:**
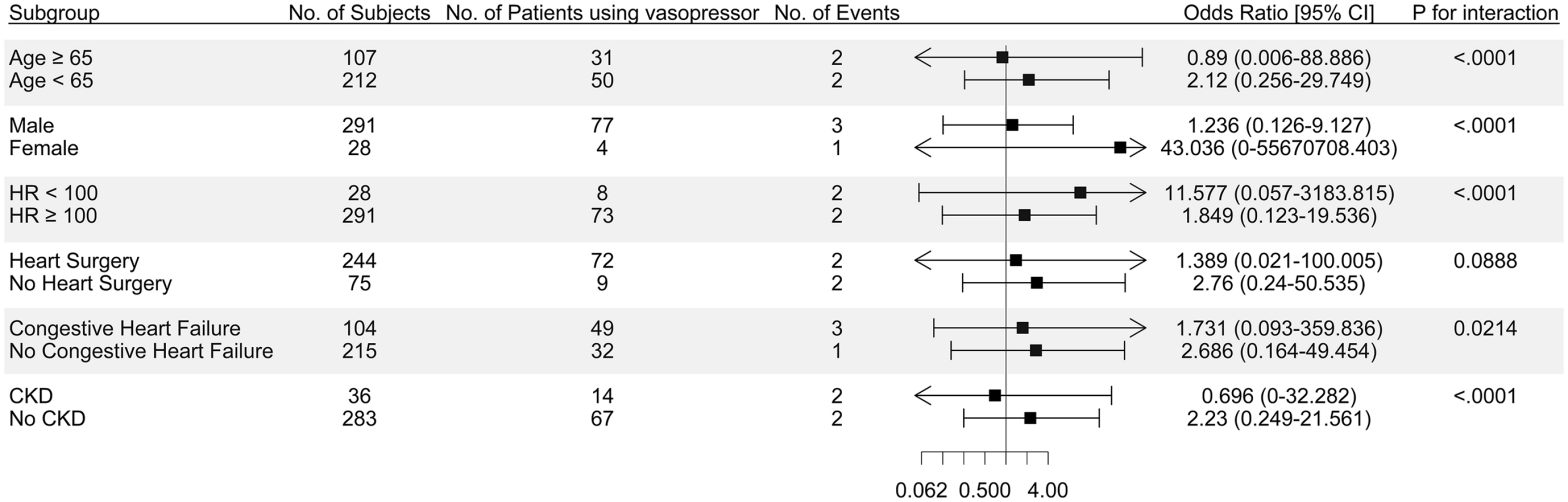
Forest plot of subgroup analysis for kidney deterioration using the fully adjusted Model 4. CI, confidence interval; HR, heart rate; CKD, chronic kidney disease.

Although interaction tests were statistically significant in all subgroups except prior heart surgery, none of the individual subgroup analyses showed a significant association between vasopressor use and kidney deterioration (Fig 2). Although the p-values for interaction terms in subgroups divided by age, sex, heart rate, congestive heart failure, and CKD were significant, vasopressor use itself was not significantly associated with kidney deterioration.

**Fig 3:**
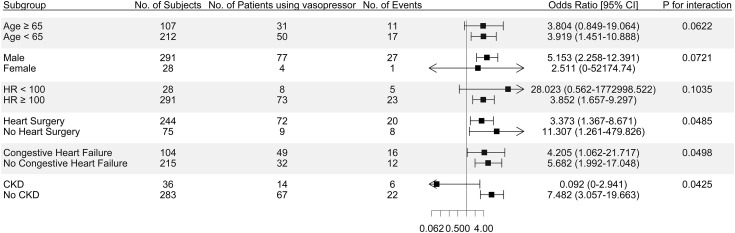
Forest plot of subgroup analysis for prolonged intensive care unit stay duration using the fully adjusted Model 4. CI, confidence interval; HR, heart rate; CKD, chronic kidney disease.

The use of vasopressors was associated with a higher risk of prolonged ICU stay duration in patients who did not undergo heart surgery compared to those who did, with a statistically significant p for interaction term (Fig 3). Additionally, in patients without congestive heart failure, the use of vasopressors was associated with a higher risk of prolonged ICU stay compared to those with congestive heart failure, with a statistically significant p for interaction term. While there was no significant association between vasopressors and prolonged ICU stay in patients with CKD, vasopressors were significantly associated with a higher risk of prolonged ICU stay in patients without CKD.

## Discussion

This study analyzed the impact of vasopressor administration on kidney deterioration and prolonged ICU stay duration in patients admitted to the ICU who were defined as having vasoplegic status (SVR < 800 dynes·s/cm^5^, CI > 2.2 L/min/m^2^, while maintaining normal blood pressure) using the large MIMIC-IV database. To our knowledge, this is the first study to evaluate the effects of vasopressors in patients with vasoplegic status who maintain normal blood pressure, providing preliminary observations distinct focusing on patients with vasoplegic shock. The results of this study showed that vasopressor use was not statistically significantly associated with kidney deterioration but was significantly related to prolonged ICU stay. Similar findings were observed in the sensitivity analysis, which grouped patients based on the type of vasopressors administered. These findings suggest that vasopressor use may be associated with longer ICU stay without clear renal benefit.

Previous studies have focused on vasoplegic shock and the effects of vasopressor administration on patient outcomes. Specifically, in cases of vasoplegia following cardiac surgery, norepinephrine or vasopressin is recommended as first-line therapy, and these agents are known to reduce mortality and improve organ perfusion [13,24,25]. Notably, vasopressin has been associated with a significantly lower incidence of adverse events such as atrial fibrillation and the need for renal replacement therapy, as shown in a recent meta-analysis [26]. Similarly, norepinephrine is recommended as first-line therapy in septic shock, where maintaining adequate blood pressure per guidelines is known to improve outcomes [27]. Epinephrine is also considered an option for treating vasoplegic shock [2,12].

However, these studies have all been conducted in patients experiencing shock, characterized by low blood pressure. The use of vasopressors can result in side effects, including increased myocardial oxygen demand, interference with cellular energy metabolism, oxidative stress, arrhythmias, and the risk of necrosis due to severe peripheral vasoconstriction [24,25,28]. Epinephrine, in particular, has been reported to worsen heart rate, increase myocardial oxygen consumption, and raise the likelihood of arrhythmias [29]. Accordingly, vasopressors should be administered with caution. Understanding whether vasopressor use is justified in patients with vasoplegic status who maintain normal blood pressure is of great importance.

Patients with vasoplegic status, despite having low SVR, are likely to maintain normal blood pressure due to high effective circulating volume and adequate cardiac output compensation [30]. This compensation may reflect increased stroke volume and reflex tachycardia—driven by sympathetic activation and enhanced venous return—which together preserve arterial pressure despite profound vasodilation. As a result, organ perfusion may already be sufficiently maintained, and increasing SVR with vasopressors to further elevate blood pressure may not enhance kidney perfusion. Instead, the use of vasopressors in this population may lead to side effects that could contribute to a longer ICU stay. Similarly, in the multicenter trial, targeting a higher MAP of 80–85 mmHg—compared with 65–70 mmHg—did not improve mortality in septic shock patients, suggesting that pushing blood pressure above conventional targets may not yield additional benefit [31]. In parallel, our findings indicate that vasopressor-induced increases in MAP above normotensive levels do not confer renal protection in patients with vasoplegic status and may instead prolong ICU stay. One possible explanation is that the inherent requirement for vasopressor dose titration and tapering in routine clinical practice may itself prolong ICU length of stay. While vasopressors may sometimes be used in normotensive patients to optimize perfusion, our findings did not demonstrate clear renal benefit and raise the possibility of prolonged ICU stay.

In the subgroup analysis of this study, although no significant association between vasopressor use and kidney deterioration was observed across all subgroups, significant interactions were found between vasopressor use and prolonged ICU stay in relation to congestive heart failure, heart surgery, and CKD. Specifically, patients without congestive heart failure, those who had not undergone heart surgery, and those without CKD showed a higher risk of prolonged ICU stay. The risk factors for adverse effects of vasopressor use in patients with normal blood pressure have not been well-studied to date. It is known that patients with congestive heart failure, those undergoing surgery, and those with CKD already exhibit increased adrenergic activity [32–34]. Therefore, vasopressors that act on alpha receptors, such as norepinephrine and epinephrine, may encounter resistance in these patient groups. Given that the patients in this study maintained normal blood pressure, there was no specific target blood pressure for vasopressor administration, making it likely that similar doses of vasopressors were administered to both patients with and without conditions like congestive heart failure, surgery, or CKD. As a result, patients without these conditions, who may have been more responsive to vasopressors, could have experienced more pronounced side effects, potentially leading to a longer ICU stay. Moreover, their greater hemodynamic responsiveness may require more cautious and prolonged vasopressor tapering, which can itself extend ICU length of stay

As this study is observational in nature, it has the inherent limitation of not being able to establish causality. Due to the retrospective design and potential selection bias, residual confounding from unmeasured variables cannot be ruled out. Additionally, since the MIMIC‑IV data were collected from a single institution, further research is needed to determine whether the findings can be generalized to other settings; moreover, known data inconsistencies may introduce bias and limit reproducibility. It is also important to note that there is no standardized definition of vasoplegic status, and the criteria used in this study may differ from those applied in other research. Additionally, CI and SVR measurements are subject to potential variability and precise timestamps linking CI, SVR, and MAP measurements to the start of vasopressor therapy were not uniformly available. Vasopressor dose and infusion duration were not captured, preventing assessment of exposure intensity. Furthermore, the overall cohort size was relatively small and the number of outcome events was limited, which may have reduced statistical power. In addition, the wide confidence intervals observed for several effect estimates reflect limited precision due to the small number of events. Despite assessment of collinearity, residual confounding cannot be excluded. Moreover, our subgroup and sensitivity analyses were exploratory—conducted in smaller subsets and with multiple comparisons—and therefore should be interpreted with caution. Future randomized controlled trials (RCTs) are needed to assess the effects of vasopressor use in patients with vasoplegic status who maintain normal blood pressure.

In conclusion, this study is the first to analyze the impact of vasopressor administration on kidney deterioration and ICU stay duration in ICU patients defined as having vasoplegic status (SVR < 800 dynes·s/cm^5^ and CI > 2.2 L/min/m^2^) while maintaining normal blood pressure. The findings revealed that while vsopressor use was not statistically associated with kidney deterioration, it was significantly associated with a prolonged ICU stay. This suggests that vasopressor use in patients with vasoplegic status may extend ICU stay without offering kidney protection. These results underscore the need for prospective studies to evaluate vasopressor use in this setting.

## Supporting information

S1 Table Concordance statistics of models. (DOCX)

S2 Table Variance inflation factors for variables. (DOCX)

S3 Table Odds ratio for kidney outcome according to using vasopressors. (DOCX)

S4 Table Odds ratio for prolonged length of intensive care unit stay according to using vasopressors. (DOCX)

S5 Table Odds ratio for kidney outcome according to using vasopressors using at a mean arterial pressure threshold of 65 mmHg. (DOCX)

S6 Table Odds ratio for prolonged length of intensive care unit stay according to using vasopressors at a mean arterial pressure threshold of 65 mmHg. (DOCX)
