# Supplementary material for: Low systemic vascular resistance with normal blood pressure: Do we need vasopressors?
Source: PLoS One. 2025 Oct 3;20(10):e0333365. doi: 10.1371/journal.pone.0333365 (PMC12494254; doi:10.1371/journal.pone.0333365)
Supplement: S1 Table — (DOCX) [file pone.0333365.s001.docx]

Supplementary Table S1. Concordance statistics of models

|  | **Concordance statistic** | |
| --- | --- | --- |
| **Model** | **Kidney outcome** | **Prolonged LOS** |
| Univariate | 0.523 | 0.572 |
| Model1 | 0.766 | 0.710 |
| Model2 | 0.960 | 0.781 |
| Model3 | 0.966 | 0.803 |
| Model4 | 0.973 | 0.821 |

Abbreviation: LOS, length of stay.
