# Supplementary material for: Low systemic vascular resistance with normal blood pressure: Do we need vasopressors?
Source: PLoS One. 2025 Oct 3;20(10):e0333365. doi: 10.1371/journal.pone.0333365 (PMC12494254; doi:10.1371/journal.pone.0333365)
Supplement: S2 Table — (DOCX) [file pone.0333365.s002.docx]

Supplementary Table S2. Variance inflation factors for variables

| **Variable** | **Variance inflation factor** |
| --- | --- |
| Vasopressors | 1.70 |
| Age | 1.54 |
| Male | 1.26 |
| SBP | 2.87 |
| DBP | 3.14 |
| Heart rate | 1.37 |
| Body surface area | 2.82 |
| Chronic kidney disease | 1.56 |
| Creatinine | 1.51 |
| Heart surgery | 2.23 |
| Aorta surgery | 1.29 |
| Initial SVR | 6.64 |
| Initial CI | 11.26 |
| SVR at low MAP | 2.42 |
| CI at low MAP | 5.06 |
| Mechanical ventilation | 1.07 |
| Atrial fibrillation | 1.25 |
| Congestive heart failure | 1.50 |
| Diabetes | 1.25 |
| Hypertension | 1.45 |
| pH | 1.53 |
| Bicarbonate | 1.53 |
| Lactate | 1.87 |
| Hemoglobin | 1.27 |

Abbreviation: SBP, systolic blood pressure; DBP, diastolic blood pressure; SVR, systemic vascular resistance; CI, cardiac index; MAP, mean arterial pressure.
