# Supplementary material for: Low systemic vascular resistance with normal blood pressure: Do we need vasopressors?
Source: PLoS One. 2025 Oct 3;20(10):e0333365. doi: 10.1371/journal.pone.0333365 (PMC12494254; doi:10.1371/journal.pone.0333365)
Supplement: S5 Table — (DOCX) [file pone.0333365.s005.docx]

Supplementary Table S5. Odds ratio for kidney outcome according to using vasopressors using at a mean arterial pressure threshold of 65 mmHg

| **Group** | **No. of group** | **No. of kidney outcome** | **Univariable** | | **Model 1^a^** | | | **Model 2^b^** | | | **Model 3^c^** | | | **Model 4^d^** | | |
| --- | --- | --- | --- | --- | --- | --- | --- | --- | --- | --- | --- | --- | --- | --- | --- | --- |
|  |  |  | **Odds ratio** | **p-value** | **Odds ratio** | **p-value** | **Odds ratio** | | **p-value** | **Odds ratio** | | **p-value** | **Odds ratio** | | **p-value** |  |
| No vasopressors | 104 | 12 | reference |  | reference |  | reference | |  | reference | |  | reference | |  |  |
| Vasopressors | 32 | 3 | 0.878 (0.214-2.831) | 0.838 | 0.728 (0.148-2.686) | 0.652 | 3.853 (0.352-123.792) | | 0.266 | 2.561 (0.108-162.236) | | 0.499 | 1.406 (0.043-93.218) | | 0.824 |  |

a: adjusted for age, male, chronic kidney disease, creatinine.

b: adjusted for age, male, systolic blood pressure, diastolic blood pressure, heart rate, body surface area, chronic kidney disease, creatinine, heart surgery, aorta surgery, initial systemic vascular resistance, initial cardiac index, systemic vascular resistance at low mean arterial pressure, cardiac index at low mean arterial pressure.

c: adjusted for age, male, systolic blood pressure, diastolic blood pressure, heart rate, body surface area, chronic kidney disease, creatinine, heart surgery, aorta surgery, initial systemic vascular resistance, initial cardiac index, systemic vascular resistance at low mean arterial pressure, cardiac index at low mean arterial pressure, mechanical ventilation, atrial fibrillation, congestive heart failure, diabetes, hypertension.

d: adjusted for age, male, systolic blood pressure, diastolic blood pressure, heart rate, body surface area, chronic kidney disease, creatinine, heart surgery, aorta surgery, initial systemic vascular resistance, initial cardiac index, systemic vascular resistance at low mean arterial pressure, cardiac index at low mean arterial pressure, mechanical ventilation, atrial fibrillation, congestive heart failure, diabetes, hypertension, ph, bicarbonate, lactate, hemoglobin.
